# Prognostic significance of combined PD-L1 expression in malignant and infiltrating cells in hepatocellular carcinoma treated with atezolizumab and bevacizumab

**DOI:** 10.3389/fimmu.2024.1506355

**Published:** 2024-12-10

**Authors:** Jaejun Lee, Jae-Sung Yoo, Ji Hoon Kim, Dong Yeup Lee, Keungmo Yang, Bohyun Kim, Joon-Il Choi, Jeong Won Jang, Jong Young Choi, Seung Kew Yoon, Ji Won Han, Pil Soo Sung

**Affiliations:** ^1^ The Catholic University Liver Research Center, Department of Biomedicine & Health Sciences, College of Medicine, The Catholic University of Republic of Korea, Seoul, Republic of Korea; ^2^ Division of Hepatology, Department of Internal Medicine, Seoul St. Mary’s Hospital, College of Medicine, The Catholic University of Republic of Korea, Seoul, Republic of Korea; ^3^ School of Medicine, Kyungpook National University, Daegu, Republic of Korea; ^4^ Division of Hepatology, Department of Internal Medicine, Uijeongbu St. Mary’s Hospital, College of Medicine, The Catholic University of Republic of Korea, Seoul, Republic of Korea; ^5^ Departmend of Radiology, Seoul St. Mary’s Hospital, College of Medicine, The Catholic University of Republc of Korea, Seoul, Republic of Korea

**Keywords:** PD-L1, HCC, atezolizumab, overall survival, objective response

## Abstract

**Background:**

Programmed death-ligand 1 (PD-L1) expression is abundant not only in malignant cells but also in infiltrating cells within the tumor microenvironment (TME) of hepatocellular carcinoma (HCC). This study explored the association between PD-L1 expression in TME and outcomes in HCC patients treated with atezolizumab plus bevacizumab (AB), emphasizing the implications of PD-L1 expression in both malignant and tumor-infiltrating cells.

**Methods:**

This study included 72 patients with HCC who underwent percutaneous core needle liver biopsy before AB treatment between September 2020 and December 2023. PD-L1 expression on tumor tissues was assessed using the combined positive score (CPS) with cutoff values of 1 and 10, utilizing antibody clone 22C3 (Dako).

**Results:**

The distribution of PD-L1 CPS included 24 patients with CPS <1, 33 patients with CPS 1–10, and 15 patients with CPS ≥10. Significant differences in overall survival (OS) were observed across the three groups, with CPS ≥10 showing the highest survival rates (p = 0.010). Patients with CPS ≥10 had better OS than those with CPS <10 (median OS 14.8 vs. 8.3 months, P = 0.046), and CPS ≥1 had better OS than CPS <1 (P = 0.021). For progression-free survival (mPFS), the CPS ≥10 group had the highest median PFS of 11.0 months among the three groups (P = 0.044). Objective response rates (ORR) were higher in the PD-L1 CPS ≥10 group than in the 1-10 and <1 group (53.3%, 27.3%, and 16.7%, respectively; *P* = .047). Multivariate analysis identified that PD-L1 expression ≥10 and ≥1 were associated with favorable outcomes regarding OS (hazard ratio [HR] 0.283, *P* = .027 and HR 0.303, *P* = .006, respectively).

**Conclusions:**

Combined analysis of PD-L1 expression in malignant and tumor-infiltrating cells can be a promising biomarker for the prognosis of HCC patients treated with AB.

## 1 Introduction

Hepatocellular carcinoma (HCC) is the most common type of primary liver cancer and ranks as the fourth leading cause of cancer-related deaths globally (1, 2). For advanced HCC, treatment options were limited to tyrosine kinase inhibitors until the recent IMbrave150 trial, which reshaped the landscape of HCC treatment through immunotherapy (3). The introduction of atezolizumab, a programmed cell death-ligand 1 (PD-L1) inhibitor, combined with bevacizumab, an anti-vascular endothelial growth factor, has significantly improved survival outcomes in patients with advanced HCC (4). Additionally, other immune checkpoint inhibitors (ICIs), such as tremelimumab plus durvalumab and nivolumab plus ipilimumab, have also been approved for HCC as first-line and second-line systemic therapies, respectively, and they have expanded the therapeutic options for clinicians treating advanced HCC (5–8). However, the objective response rate (ORR) of these ICIs remains mostly under 30%, highlighting the need for promising biomarkers to identify patients who could benefit the most from these treatments (3, 5, 6).

The programmed cell death protein 1 (PD-1)/PD-L1 axis plays a crucial role in the immune evasion mechanisms of tumors. PD-L1 on tumor cells binds to PD-1 on T cells, leading to the inhibition of T cell function and allowing the tumor to evade the immune response (9). Moreover, recent studies have highlighted the role of PD-L1 expression not only on tumor cells but also on tumor-infiltrating cells such as tumor-associated macrophages (TAMs), which are pivotal in regulating anti-tumor immunity (10–12). Given that ICIs primarily target PD-1/PD-L1 axis, numerous studies have investigated the implications of PD-L1 expression levels across various malignancies when treating these patients with ICIs (13–15). For most of the studies, consistent results were observed regarding better treatment outcomes for those with higher PD-L1 expression than those with lower expression (16, 17).

Since the advent of atezolizumab plus bevacizumab (AB), which has significantly impacted the treatment landscape of HCC, many studies have been conducted to identify biomarkers that can predict the outcome of ICIs in advanced HCC. PD-L1 is one of the most promising candidates for predictive markers (18, 19). However, the role of PD-L1 as a predictive marker has not been clearly established in HCC. Clinical trials such as CheckMate 459, KEYNOTE-224, and CheckMate 040 have presented consistent results supporting PD-L1 expression as a favorable biomarker for ICI-treated HCC (6, 20, 21). In contrast, results from studies such as the HIMALAYA trial have shown that the efficacy of these drugs is independent of PD-L1 expression status (5, 22, 23).

While controversy exists over whether PD-L1 expression levels can serve as a biomarker for ICI-treated HCC, it is important to note that previous clinical trials have been conducted across various nations and institutions, resulting in inconsistent procedures for detecting PD-L1. Moreover, the majority of these studies used tumor proportion score (TPS), which counts PD-L1 expression only in tumor cells, thus providing a limited evaluation of the tumor microenvironment (TME). In this study, we aimed to address these concerns by comparing the outcomes of patients with advanced HCC treated with AB, stratified by diverse thresholds of PD-L1 expression levels in both malignant and tumor-infiltrating cells, using a reliable and uniform antibody clone for detecting PD-L1.

## 2 Methods

### 2.1 Patients

In this study, we retrospectively reviewed the medical records of patients with unresectable HCC who received AB treatment between September 2020 and December 2023. The diagnosis of HCC was based on either histological or radiological examinations such as computed tomography or, magnetic resonance imaging, or all three. The inclusion criteria were as follows: 1) patients diagnosed with unresectable HCC; 2) availability of histological data with immunohistochemical staining for PD-L1 in cells (malignant and tumor-infiltrating cells) obtained via liver biopsy prior to the initiation of AB treatment; 3) age ≥ 18 years; 4) Eastern Cooperative Oncology Group (ECOG) performance status ≤ 1; and 5) patients who had at least one follow-up visit at the clinic after receiving AB treatment. Patients with concurrent extrahepatic malignancies or severe liver dysfunction classified as Child-Pugh class C were excluded from the study (**Supplementary Table S1**). This study was approved by the Institutional Review Board of the Catholic University of Korea (approval number: KC22EASI0342) and was performed in accordance with the Declaration of Helsinki. Informed consent was waived due to the retrospective nature of the study.

### 2.2 Treatment protocols and response evaluation

AB was administered following the standard dosing regimen outlined in the IMbrave 150 trial, which involved intravenous doses of 1200 mg of atezolizumab and 15 mg/kg of bevacizumab every three weeks. Tumor response was assessed approximately every three to four treatment cycles using the mRECIST (modified Response Evaluation Criteria in Solid Tumors) criteria (24). Treatment response was evaluated in all patients using follow-up liver dynamic computed tomography (CT) or dynamic magnetic resonance imaging (MRI) with liver-specific contrast agents. According to the mRECIST criteria, disease progression was defined as an increase in the diameter of the viable lesion by more than 20%. Treatment with AB was continued until disease progression, death, or the occurrence of intolerable adverse events.

### 2.3 Assessment of PD-L1 expression level

PD-L1 expression was assessed using the 22C3 antibody clone (1:50 dilution, Cat# M3653, Dako), which is used for the detection of the extracellular epitope (25). First, a tumor sample was obtained via core-needle liver biopsy. A 4-µm thick cross-section of the paraffin-embedded block was placed on a glass slide. Deparaffinization, rehydration, and antigen retrieval were performed using the CC1 antigen retrieval solution (Ventana Medical Systems) in an automated slide stainer (Ventana Medical Systems) for 64 minutes at 95-100°C. The sample was incubated with the 22C3 antibody for 32 minutes at 37°C and then washed with phosphate-buffered saline. After washing, the EnVision+ system HRP-labeled polymer (Dako) was applied to the slides at 24°C for 5 minutes. The slides were then treated with 3,3′-diaminobenzidine for 5 minutes and counterstained with hematoxylin. Finally, sections were dehydrated, cleared, and mounted for microscopic examination.

In the present study, a combined positive score (CPS) was used to quantify PD-L1 expression levels (26). CPS was determined by summing the number of viable PD-L1-positive tumor cells and the number of positive tumor-infiltrating cells, such as lymphocytes and macrophages, and then dividing that total by the overall number of viable tumor cells, with a maximum score of 100. Two different cut-off levels of CPS 1 and 10, based on previous studies, were employed to assess the outcomes of AB treatment in relation to PD-L1 expression levels (27).

### 2.4 Study endpoints

The primary endpoint of the study was overall survival (OS), defined as the duration between the start of AB treatment and death from any cause. Patients who were lost to follow-up or remained alive at the end of the study period were considered censored. Secondary endpoints were progression-free survival (PFS) and objective response rate (ORR). PFS was defined as the period from the start of the AB treatment until disease progression or death. ORR was defined as the proportion sum of complete response (CR) and partial response (PR), according to the mRECIST criteria.

### 2.5 Statistical analysis

All analyses were performed using the R statistical software (version 4.0.3; R Foundation Inc., Vienna, Austria; http://cran.r-project.org, accessed on June 10, 2024). Continuous variables are reported as mean values with standard deviations. Student t-test was performed when continuous variables for two independent group were compared while an analysis of variance was performed to compare among groups of three or more. Categorical variables were assessed with the chi-square test. Survival analyses were conducted via the Kaplan–Meier method, with differences evaluated using the log-rank test. Cox regression analyses were utilized to identify factors associated with survival outcomes, with those showing P <.20 in univariate analysis included in multivariate analysis. The time-dependent area under the curve of receiver operating characteristic (AUC-ROC) was utilized to assess the predictive performance of PD-L1 expression levels for survival outcomes. A restricted cubic spline was applied to estimate the trend of the dose-response relationship between PD-L1 expression levels and survival outcomes, such as OS and PFS. Statistical significance was determined at P <.05.

## 3 Results

### 3.1 Baseline characteristics

A total of 72 patients were included in the study. **Table 1** shows the baseline characteristics of the study population. Males were predominant (84.7%), and the mean age of the study population was 62.1 years. The most common cause of HCC was Hepatitis B virus infection (56.9%) followed by alcoholic liver disease (27.8%). Regarding liver function, 61.1% had a Child-Pugh score of 5, 22.2% had a Child-Pugh score of 6, and 16.7% had a Child-Pugh score of 7. Additionally, 31.9% of the patients had a single tumor mass, and the mean tumor size was 7.6 cm. In terms of tumor stage, the majority of the study population had advanced HCC, with 54.2% and 93.1% exhibiting mUICC stage IVB and BCLC stage C, respectively. The mean AFP level and PIVKA level were 628.0 ng/mL and 1298.0 mAU/mL, respectively.

**Table 1:** Baseline characteristics of enrolled patients.

	Total (n=72)	PD-L1 (CPS<1) (n=24)	PD-L1 (CPS 1–10) (n=33)	PD-L1 (CPS≥10) (n=15)	P
Male sex	61 (84.7)	19 (79.2)	30 (90.9)	12 (80.0)	0.405
Age	62.1 ± 11.7	60.2 ± 9.1	60.4 ± 13.3	68.8 ± 9.6	0.042
Etiology					0.328
HBV	41 (56.9)	15 (62.5)	20 (60.6)	6 (40.0)	
HCV	5 (6.9)	1 (4.2)	3 (9.1)	1 (6.7)	
Alcohol	20 (27.8)	7 (29.2)	8 (24.2)	5 (33.3)	
Others	6 (8.3)	1 (4.2)	2 (6.1)	3 (20.0)	
PLT (10^9^/ L)	176.0 ± 94.6	187.7 ± 115.5	176.4 ± 84.0	151.9 ± 80.1	0.513
AST (IU/L)	76.4 ± 84.8	109.0 ± 122.6	57.1 ± 52.2	66.5 ± 52.3	0.063
ALT (IU/L)	33.4 ± 21.9	40.0 ± 27.1	29.6 ± 16.5	31.2 ± 22.1	0.193
TB (mg/dL)	0.9 ± 0.6	0.9 ± 0.5	1.0 ± 0.7	0.8 ± 0.5	0.791
Albumin (mg/dL)	3.8 ± 0.4	3.7 ± 0.4	3.8 ± 0.5	3.8 ± 0.4	0.715
PT (INR)	1.1 ± 0.1	1.1 ± 0.1	1.1 ± 0.1	1.2 ± 0.2	0.981
Child-Pugh class					0.961
A5	44 (61.1)	14 (58.3)	20 (60.6)	10 (66.7)	
A6	16 (22.2)	5 (20.8)	8 (24.2)	3 (20.0)	
B7	12 (16.7)	5 (20.8)	5 (15.2)	2 (13.3)	
Tumor no.(single)	23 (31.9)	4 (16.7)	15 (45.5)	4 (26.7)	0.063
Tumor size (cm)	7.6 ± 5.5	8.5 ± 6.6	6.7 ± 4.7	8.0 ± 5.4	0.455
mUICC stage					0.710
II	2 (2.8)	1 (4.2)	1 (3.0)	0 (0.0)	
III	4 (5.6)	1 (4.2)	3 (9.1)	0 (0.0)	
IVa	27 (37.5)	8 (33.3)	11 (33.3)	8 (53.3)	
IVb	39 (54.2)	14 (58.3)	18 (54.5)	7 (46.7)	
BCLC stage					0.490
B/C	5 (6.9)/67 (93.1)	2 (8.3)/22 (91.7)	3 (9.1)/30 (90.9)	0 (0.0)/15 (100.0)	
AFP (ng/mL)	628.0 (18.6, 19259.5)	1171.5 (19.5, 23676.5)	887.0 (18.6, 18462.0)	457.0 (23.7, 3836.5)	0.858
PIVKA (mAU/mL)	1298.0 (175.0, 7783.0)	5193.0 (419.5, 19482.8)	1045.0 (123.0, 3264.0)	772.0 (113.0, 8046.0)	0.261

Values are presented as mean ± standard deviation or number (%). AFP, alpha fetoprotein; AST, aspartate aminotransferase; ALT, alanine aminotransferase; BCLC, Barcelona clinic liver cancer; CPS, combined positive score; HBV, hepatitis B virus; HCV, hepatitis C virus; mUICC, modified union for international cancer control; PIVKA, protein induced by vitamin K absence; PLT, platelet; PT, prothrombin time; TB, total bilirubin.

The study population was categorized into three groups according to PD-L1 expression levels using CPS cutoff values of 1 and 10. The three groups had no differences in sex distribution; however, the high PD-L1 (CPS ≥10) group was older than the other groups (*P* = .042).

### 3.2 Representative immunohistochemical findings of the enrolled patients

**Figure 1:**
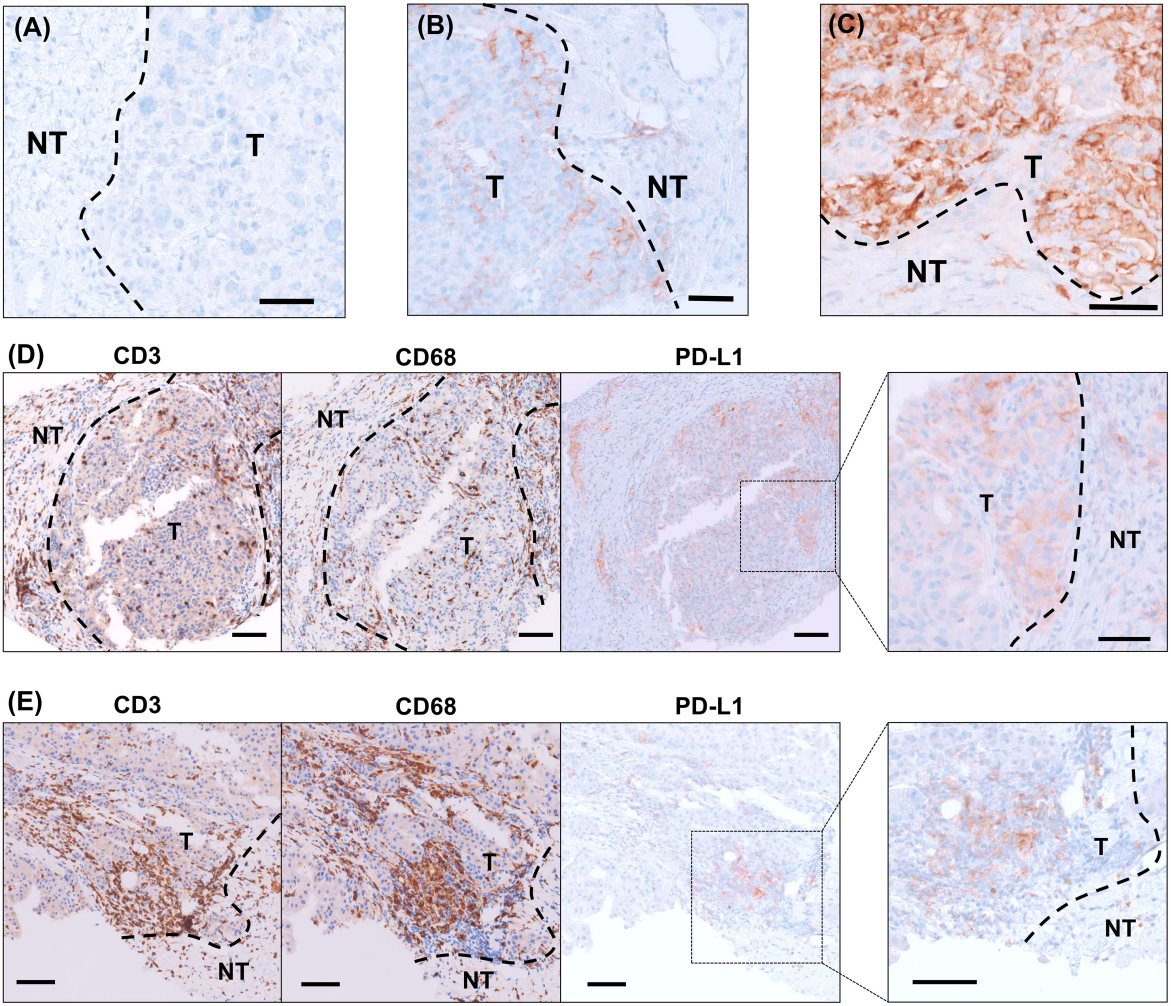
Representative images of PD-L1 immunohistochemistry in the biopsy samples. **(A)** PD-L1 immunohistochemical staining in a biopsy sample from a patient with low PD-L1 (CPS <1) expression, who experienced disease progression within 2 months. **(B)** PD-L1 immunohistochemical staining in a biopsy sample from a patient with a PD-L1 CPS of 5, who achieved progression-free survival for more than 12 months. **(C)** PD-L1 immunohistochemical staining in a biopsy sample from a patient with high PD-L1 expression (CPS 90), who achieved a partial response following treatment with atezolizumab plus bevacizumab. **(D)** Immunohistochemical staining of tumor tissues for CD3, CD68, and PD-L1, with PD-L1 staining predominantly positive in malignant cells. **(E)** Immunohistochemical staining for CD3, CD68, and PD-L1, with PD-L1 staining primarily positive in tumor-infiltrating non-malignant cells. Scale bar represents 100 µm; CPS, combined positive score; NT, non-tumor; T, tumor.

**Figure 1** displays the representative immunohistochemical findings for the enrolled patients, including samples with high or low PD-L1 expression and diverse prognoses. PD-L1 immunohistochemistry slides (**Figures 1A–C**) show varying PD-L1 expression levels (CPS <1, 5, 90) in biopsy samples from patients with different clinical outcomes. In addition, PD-L1 expression in malignant cells versus tumor-infiltrating non-malignant cells is also presented in **Figures 1D, E**.

### 3.3 Treatment responses

Tumor response to AB treatment was assessed (**Table 2**). Overall, 21 patients achieved partial response, resulting in an ORR of 29.2%. Additionally, 21 patients achieved stable disease, resulting in a disease control rate (DCR) of 58.4%. Twenty-two patients exhibited progressive disease after AB treatment and radiologic assessments were not performed in eight patients during the treatment.

**Table 2:** Treatment responses by PD-L1 expression level.

	PD-L1<1 (n = 24)	PD-L1 1–10 (n = 33)	PD-L1≥10 (n = 15)	*P* value
Treatment responses				0.105
PR	4 (16.7)	9 (27.3)	8 (53.3)	
SD	7 (29.2)	10 (30.3)	4 (26.7)	
PD	11 (45.8)	8 (24.2)	3 (20.0)	
NA	2 (8.3)	6 (18.2)	0 (0.0)	
ORR	4 (16.7)	9 (27.3)	8 (53.3)	0.047
DCR	11 (45.8)	19 (57.6)	12 (80.0)	0.108

Values are presented as number (%). DCR, disease control rate; ORR, objective response rate; PD, progressive disease; PR, partial response; SD, stable disease, NA, not applicable.

The three groups stratified by PD-L1 expression levels were compared with respect to treatment response (**Table 2**). For ORR, the high PD-L1 (CPS ≥10) group had the highest rate at 53.3% (n = 8), followed by the intermediate PD-L1 (CPS 1-10) group at 27.3% (n = 9), and the low PD-L1 (CPS <1) group at 16.7% (n = 4) (*P* = .047). Regarding DCR, the high PD-L1 group had the highest rate at 80.0% compared with the intermediate PD-L1 group (57.6%) and the low PD-L1 group (45.8%), although the difference was not statistically significant (*P* = .108). Next, CPS 1 and 10 were each applied as a sole cutoff value and assessed for tumor response rates. For ORR, the high PD-L1 group showed higher ORR compared to the low PD-L1 group (CPS ≥10: 53.3% vs. CPS <10: 22.8%, *P* = .021; CPS ≥1: 35.4% vs. CPS <1: 16.7%, *P* = .099). In terms of DCR, the high PD-L1 groups were higher than the low PD-L1 groups, although statistical significance was not reached (CPS ≥10: 80.0% vs. CPS <10: 52.6%, *P* = .055; CPS ≥1: 64.6% vs. CPS <1: 45.8%, *P* = .128).

### 3.4 Overall survival based on the PD-L1 expression levels

**Figure 2:**
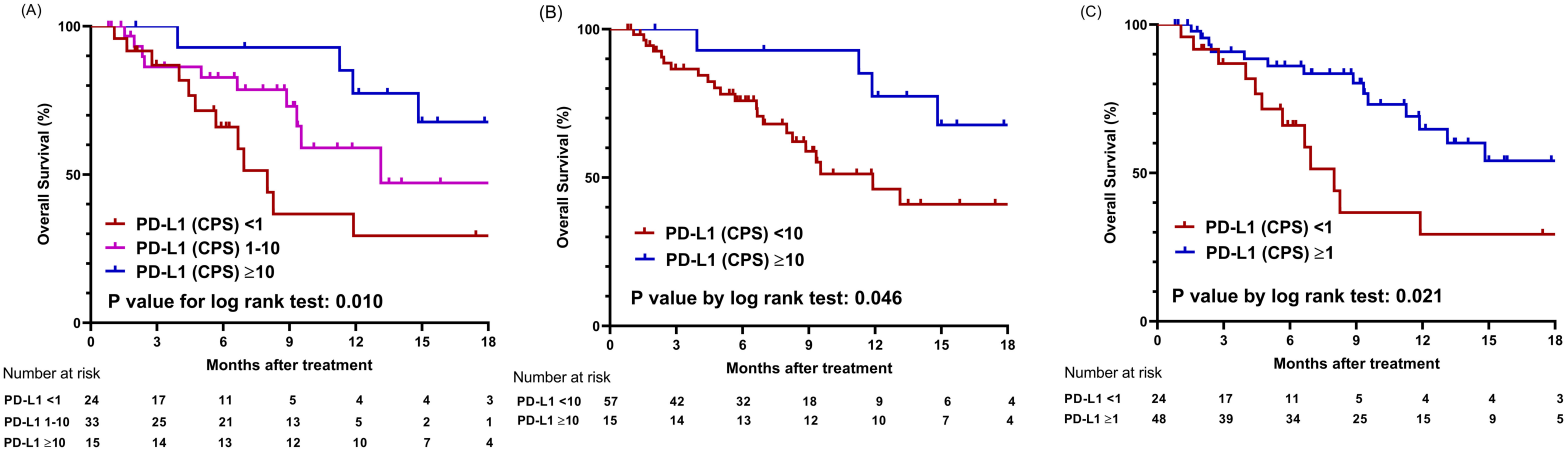
Comparison of overall survival based on PD-L1 expression levels using different cut-off values. **(A)** Graphs showing comparison of overall survival among three groups categorized by cutoff values of CPS 1 and 10. **(B)** Graphs showing comparison of overall survival between two groups categorized by cutoff value of CPS 10. **(C)** Graphs showing comparison of overall survival between two groups categorized by cutoff value of CPS 1; CPS, combined positive score.

During the median follow-up period of 7.4 months, 26 mortality cases were documented. The median overall survival (mOS) for the entire cohort was 14.8 months (95% confidence interval [CI], 11.3 months–NA). First, using CPS values of 1 and 10 as cutoff points, patients were categorized into three groups and compared for OS (**Figure 2A**). The OS was significantly longer in the high PD-L1 (CPS ≥10) group, with 92.9% survival rates at six months and 77.4% survival rates at 12 months, compared to the intermediate PD-L1 (CPS 1-10) group (mOS 13.1 months) and the low PD-L1 (CPS <1) group (mOS 8.0 months) (*P* = .010). When comparing the two groups based on a single PD-L1 CPS cutoff of 10, the high PD-L1 (CPS ≥10) group again exhibited better OS than the low PD-L1 (CPS <10) group (mOS 11.9 months) (*P* = .046) (**Figure 2B**). Using a PD-L1 CPS of 1 as a cutoff value, the 6-month and 12-month survival rates of the high PD-L1 (CPS ≥1) group were 86.1% and 64.7%, respectively, which were higher than the 66.0% and 29.3% survival rates of the low PD-L1 (CPS <1) group (*P* = .021) (**Figure 2C**).

### 3.5 Progression-free survival based on the PD-L1 expression levels

**Figure 3:**
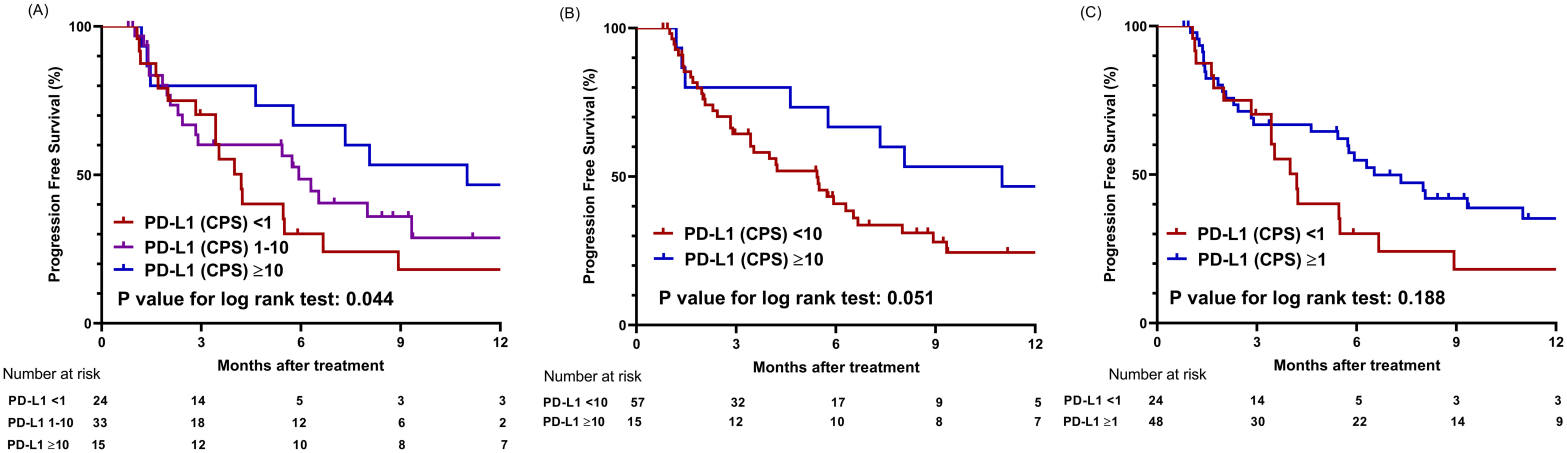
Comparison of progression-free survival based on PD-L1 expression levels using different cut-off values. **(A)** Graphs showing comparison of progression-free survival among three groups categorized by cutoff values of CPS 1 and 10. **(B)** Graphs showing comparison of progression-free survival between two groups categorized by cutoff value of CPS 10. **(C)** Graphs showing comparison of progression-free survival between two groups categorized by cutoff value of CPS 1; CPS, combined positive score.

The median progression-free survival (mPFS) for the entire cohort was 5.8 months (95% CI, 4.2–8.9 months). When the study populations were stratified into three groups using CPS values of 1 and 10 as cutoff points, the high PD-L1 group showed the longest mPFS of 11.0 months (95% CI, 5.8 months–NA) compared to the intermediate PD-L1 group (mPFS 5.9 months, 95% CI, 2.8 months–NA) and the low PD-L1 group (mPFS 4.0 months, 95% CI, 3.4–8.9 months) (*P* = .044) (**Figure 3A**). When CPS 10 was used as the sole cut-off value, the high PD-L1 group (mPFS not applicable (NA)) showed a tendency toward longer PFS than the low PD-L1 group (mPFS 5.43 months) (*P* = .051) (**Figure 3B**). When CPS 1 was employed as a cut-off value, the mPFS for the high PD-L1 and low PD-L1 groups were 4.6 months (95% CI, 2.1–7.1 months) and 2.8 months (95% CI, 2.2–6.6 months), respectively (*P* = .188) (**Figure 3C**).

Regarding dose-dependent correlation between PFS and PD-L1 expression level, the hazard ratio (HR) for progression or death tended to decline as the PD-L1 expression level increased, although this trend was not statistically significant (*P* = .146) (**Supplementary Figure S1B**). For PD-L1 CPS of 10, the HR for PFS was 0.77 (95% CI 0.58–1.02).

### 3.6 Factors contributing to survival outcomes

Various factors that could affect the survival outcomes were included in the analysis. In terms of OS, univariate analysis revealed that ECOG 1 and a Child-Pugh score of 5 were associated with the outcomes. Subsequently, factors with a *P* value of less than 0.2 in the univariate analysis were included in the multivariate analysis. Two models were assessed, each incorporating different cutoff values for CPS as a marker for PD-L1 expression. In Model 1, which used PD-L1 (CPS ≥10) as the biomarker, PD-L1 (CPS ≥10) was the only factor associated with OS (HR 0.283, 95% CI, 0.092–0.865; *P* = .027). In Model 2, which used PD-L1 (CPS ≥1) as the covariate, PD-L1 (CPS ≥1) remained the only significant factor favorably associated with OS (HR 0.303, 95% CI, 0.128–0.713; *P* = .006) (**Table 3**).

**Table 3:** Factors associated with survival outcomes.

	Univariate analysis		Multivariate analysis			
			Model 1		Model 2	
	HR (95% CI)	*P* value	HR (95% CI)	*P* value	HR (95% CI)	*P* value
PD-L1 ≥ 10 CPS	0.346 (0.117, 1.024)	0.055	0.283 (0.092, 0.865)	0.027	–	–
PD-L1 ≥ 1 CPS	0.409 (0.187, 0.893)	0.025	–	–	0.303 (0.128, 0.713)	0.006
Sex (Female)	1.397 (0.525, 3.715)	0.503				
Age≥65	1.147 (0.531, 2.481)	0.727				
ECOG 1 (vs. 0)	2.342 (1.078, 5.090)	0.032	2.162 (0.916, 5.101)	0.078	2.293 (0.959, 5.482)	0.062
Etiology-Viral (vs non-viral)	0.846 (0.383, 1.868)	0.678				
Child-Pugh score 5	0.449 (0.207, 0.974)	0.043	0.510 (0.224, 1.162)	0.109	0.522 (0.227, 1.201)	0.126
AFP>400ng/mL	1.619 (0.747, 3.508)	0.222				
Tumor size>5cm	2.101 (0.928, 4.756)	0.075	1.657 (0.687, 3.999)	0.261	1.820 (0.759, 4.367)	0.180
Number of tumors≥2	1.082 (0.482, 2.433)	0.848				
Vascular invasion	0.659 (0.305, 1.422)	0.287				
Extrahepatic metastasis	2.084 (0.925, 4.695)	0.076	1.465 (0.618, 3.475)	0.386	1.514 (0.633, 3.618)	0.351

Model 1 includes PD-L1 ≥ 10 CPS as a covariate, and Model 2 includes PD-L1 ≥ 1 CPS as a covariate. AFP, alpha fetoprotein; CI, confidence interval; ECOG, Eastern Cooperative Oncology Group; HR, hazard ratio.

Regarding PFS, only a Child-Pugh score of 5 remained significant in the univariate analysis. In multivariate analysis Model 1, which included PD-L1 (CPS ≥10), sex, ECOG score, Child-Pugh score, and extrahepatic metastasis as variables, PD-L1 (CPS ≥10) (HR 0.406, 95% CI 0.188–0.878, *P* = .022), female sex (HR 2.643, 95% CI 1.146–6.096, *P* = .023), and Child-Pugh score 5 (HR 0.503, 95% CI 0.269–0.941, *P* = .032) were significantly associated with PFS in the study cohort. In Model 2, female sex (HR 2.339, 95% CI 1.018–5.373, *P* = .045) and Child-Pugh score 5 (HR 0.525, 95% CI 0.282–0.977, *P* = .042) were associated with PFS (**Table 4**).

**Table 4:** Factors associated with progression-free survival.

	Univariate analysis		Multivariate analysis			
			Model 1		Model 2	
	HR (95% CI)	*P* value	HR (95% CI)	*P* value	HR (95% CI)	*P* value
PD-L1 ≥ 10 CPS	0.493 (0.240, 1.013)	0.054	0.406 (0.188, 0.878)	0.022	–	–
PD-L1 ≥ 1 CPS	0.675 (0.375, 1.214)	0.189	–	–	0.583 (0.317, 1.071)	0.082
Sex (Female)	1.697 (0.792, 3.635)	0.173	2.643 (1.146, 6.096)	0.023	2.339 (1.018, 5.373)	0.045
Age≥65	0.806 (0.457, 1.423)	0.458				
ECOG 1 (vs. 0)	1.532 (0.870, 2.696)	0.140	1.249 (0.683, 2.283)	0.470	1.201 (0.651, 2.215)	0.557
Etiology-Viral (vs non-viral)	1.251 (0.687, 2.278)	0.463				
Child-Pugh score 5	0.552 (0.311, 0.979)	0.042	0.503 (0.269, 0.941)	0.032	0.525 (0.282, 0.977)	0.042
AFP>400ng/mL	1.306 (0.743, 2.296)	0.354				
Tumor size>5cm	1.345 (0.757, 2.389)	0.312				
Number of tumors≥2	1.113 (0.612, 2.026)	0.726				
Vascular invasion	0.739 (0.415, 1.315)	0.303				
Extrahepatic metastasis	1.728 (0.968, 3.084)	0.064	1.401 (0.762, 2.573)	0.278	1.593 (0.867, 2.928)	0.134

Model 1 includes PD-L1 ≥ 10 CPS as a covariate, and Model 2 includes PD-L1 ≥ 1 CPS as a covariate. AFP, alpha fetoprotein; CI, confidence interval; ECOG, Eastern Cooperative Oncology Group; HR, hazard ratio.

### 3.7 Predictive performance of PD-L1 on survival outcomes

**Figure 4:**
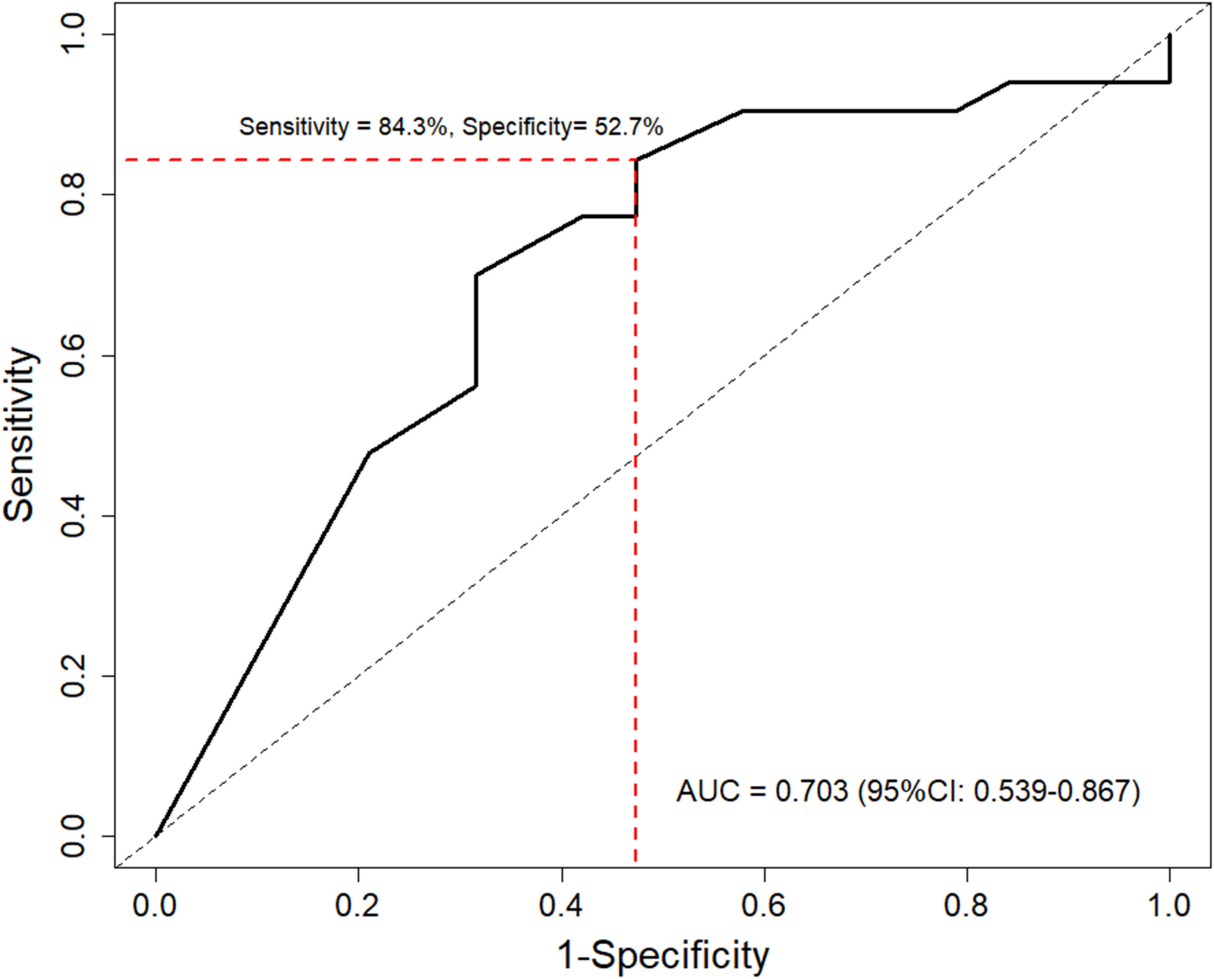
Time-dependent receiver operating characteristic curve of PD-L1 levels for predicting 12-month overall survival. Red line indicates optimal cutoff value determined by Youden’s index. AUC, area under the curve.

To evaluate the predictive performance of PD-L1 on survival outcomes, the time-dependent AUC-ROC was calculated for 12-month OS (**Figure 4**). The AUC-ROC for 12-month OS in the study population was 0.703 (95% CI: 0.539–0.867). Using Youden’s index, a CPS of 5 was identified as the optimal cutoff value for predicting survival outcomes, with a sensitivity of 84.3% and specificity of 52.7%. Furthermore, the HR for survival outcomes was assessed based on PD-L1 expression level. The HR tended to decline as PD-L1 expression level increased (*P* = .031) (**Supplementary Figure S1A**). Using a CPS of 5 as a reference, the HR was 0.60 (95% CI 0.38–0.94) for CPS 10 of the PD-L1 level.

### 3.8 Sensitivity analysis using different cutoff value for PD-L1 expression

A PD-L1 CPS of 5 was used as an alternative cutoff value to perform a sensitivity analysis on the impact of PD-L1 expression levels on survival outcomes. For OS, the high PD-L1 (CPS ≥5) group exhibited 6- and 12-month survival rates of 85.7% and 71.9%, respectively, which were significantly higher than the 76.6% and 43.0% survival rates of the low PD-L1 (CPS < 5) group (*P* = .044) (**Supplementary Figure S2A**). In terms of PFS, the high PD-L1 group (mPFS 6.5 months, 95% CI, 1.6 months–NA) tended towards prolonged PFS compared to the low PD-L1 group (mPFS 3.4 months, 95% CI, 2.2–5.3 months) (*P* = .069) (**Supplementary Figure S2B**).

### 3.9 Subgroup analysis in patients with viral etiologies

Survival outcomes were assessed in patients with viral etiologies (n = 46). For OS, patients with higher PD-L1 expression exhibited longer survival times, although the difference was not statistically significant (mOS: CPS ≥1 NA, CPS 1–10 13.1 months, and CPS <1 8.0 months, *P* = .095) (**Supplementary Figure S3A**). In terms of PFS (**Supplementary Figure S3B**), patients with PD-L1(CPS ≥10) exhibited a mPFS of 17.2 months, which was higher than CPS 1-10 (mPFS 5.9 months) and CPS<1 group (mPFS 3.4 months) (*P* = .173).

## 4 Discussion

In patients with advanced HCC, AB treatment is considered first-line systemic therapy. However, as the IMbrave150 trial demonstrated, only about 30% of patients exhibit a tumor response to AB, highlighting the need for effective biomarkers to identify those who will benefit most from this treatment (28). Unfortunately, no specific biomarker for this identification has been established to date. Our study revealed that patients with high PD-L1 expression levels in malignant and tumor-infiltrating cells showed favorable outcomes in terms of both OS and PFS. Specifically, a PD-L1 level with a CPS of 10 or higher was identified as a good prognostic factor for these patients in terms of both OS and PFS. Moreover, the tumor response rate was higher in tumors with high PD-L1 expression than in those with intermediate or low PD-L1 expression. Overall, present study meticulously elucidated the impact of PD-L1 expression on survival outcomes in patients with HCC treated with AB.

The TME of HCC is characterized by a complex interplay between various cellular components, among which TAMs, cancer-associated fibroblasts, and other tumor-infiltrating immune cells play a crucial role in modulating antitumor immunity (29, 30). In addition, TAMs and other immune cells, such as dendritic cells and regulatory T cells, express PD-L1, contributing significantly to the immunosuppressive nature of the TME (31). By expressing PD-L1, these cells inhibit the cytotoxic functions of CD8+ T cells and enhance the activity of regulatory T cells, thus creating an environment favorable to tumor growth and progression​. In this context, it has been proposed that patients with high PD-L1 expression in tumor-infiltrating cells might benefit more from ICIs than those with lower expression levels (32–34). While numerous studies on various types of malignancies have demonstrated a correlation between PD-L1 expression levels and treatment outcomes, relatively few studies have explored this correlation specifically in HCC (16, 17). Among these studies, different outcomes have been observed. Regarding ORR, a study using the CheckMate 459 trial demonstrated a superior outcome for tumors with TPS of PD-L1 ≥1% compared to those with PD-L1 <1% (28% vs 12%) (35). In terms of survival outcomes, such as OS, a study utilizing the CheckMate 040 cohort showed improved OS for tumors with PD-L1 ≥1% in tumor cells compared to those with PD-L1 <1%, which is consistent with the results from our study (36). However, other studies have shown insignificant differences in ORR and survival outcomes between PD-L1 positive and negative tumors, raising controversies regarding this issue (22, 37–39).

Several factors can explain these differences between the studies. First, the use of different PD-L1 immunohistochemistry assays between studies might result in inter-assay variation, causing heterogeneity in study results (40). To date, there are five Food and Drug Administration-approved diagnostic assays for PD-L1 detection, including 22C3, SP142 (Ventana), SP263 (Ventana), 28-8 (Dako), and 73-10 (Dako) (41). Among these diagnostic assays, 22C3 is utilized across a variety of tumor types with various cutoff values. Additionally, 22C3 is approved as a companion diagnostic assay for non-small cell lung cancer, gastric cancer, cervical cancer, urothelial carcinoma, and head and neck squamous cell carcinoma, whereas the other assays serve as complementary diagnostic tests (41, 42). Notably, 22C3 has demonstrated superior sensitivity compared to other assays and has been shown to correlate well with the tumor immune microenvironment in HCC, enhancing the reliability of results obtained using this method (43, 44). Our study’s use of 22C3 exclusively may contribute to the robustness of our findings. Furthermore, previous studies have employed diverse treatment modalities, which might have contributed to the heterogeneity between the studies. In this context, the results derived from diverse settings cannot be directly applied to patients with HCC treated with AB. Thus, the results of our study hold the importance for the implication of PD-L1 expression in AB-treated HCC. Lastly, variations in counting methods for defining PD-L1 expression could account for discrepancies among studies. Our study carefully counted PD-L1 staining cells not only in malignant cells but also in tumor-infiltrating cells such as macrophages and lymphocytes. Given the critical role of TAMs and other tumor-infiltrating cells in the TME, including tumor-infiltrating cells expressing PD-L1 is essential for accurately reflecting the immunological context within tumors (45). This comprehensive approach provides a more integrated view of the tumor immune environment, which may lead to more accurate predictions of treatment response.

Our study focused on the predictive performance of PD-L1 levels for 12-month OS in HCC patients treated with AB. The results showed that the AUC-ROC was 0.703, indicating good performance of PD-L1 as a biomarker. We also assessed the dose-dependent relationship between PD-L1 expression and survival outcomes. In a restricted cubic spline curve analysis, a clear tendency of decreasing HR for survival outcomes with increasing PD-L1 level was observed. Moreover, consistent results favoring high PD-L1 expression for survival outcomes were observed in analyses using different cutoff values, namely 1, 5, and 10. In this context, our study results indicate that regardless of the definition of PD-L1 positivity, PD-L1 expression is associated with a good prognosis in HCC patients treated with AB.

While our research provides valuable insights, it also has several limitations. First, the retrospective design necessitates further investigation using a prospective design to enhance the evidence level of our results. Another limitation is the relatively small sample size collected from a single center. Additionally, the lack of data on immune cell populations and cytokine profiles restricts the comprehensive understanding of the mechanisms underlying our findings. Future studies incorporating these profiles before and after AB treatment would improve our understanding of the pathophysiological implications of our analysis. Another limitation lies in the invasiveness of biopsy procedures, which may limit the practical accessibility of PD-L1 expression as a biomarker in real-world clinical settings. Lastly, the majority of our study population had hepatitis B virus infection as the etiology of HCC. Given the potential differences in immune contexture between viral and non-viral etiologies of HCC, validation including patients with non-viral HCC is warranted (46, 47).

Through meticulous analysis, PD-L1 expression levels in malignant and tumor-infiltrating cells were identified as prognostic factors in patients with HCC treated with AB. This finding highlights the potential of PD-L1 expression levels as a biomarker for these patients. As patients with high PD-L1 expression exhibited promising survival outcomes, those in this category may be particularly suitable candidates for AB treatment. Conversely, clinicians might consider alternative treatments for tumors with low or no PD-L1 expression (48). Additionally, performing immunohistochemistry on liver biopsy specimens before selecting a treatment modality could guide clinicians in making more informed choices, potentially leading to improved treatment outcomes.

## Data Availability

The raw data supporting the conclusions of this article will be made available by the authors, without undue reservation.
