# Supplementary material for: Prognostic significance of combined PD-L1 expression in malignant and infiltrating cells in hepatocellular carcinoma treated with atezolizumab and bevacizumab
Source: Front Immunol. 2024 Dec 10;15:1506355. doi: 10.3389/fimmu.2024.1506355 (PMC11666515; doi:10.3389/fimmu.2024.1506355)

**Supplemental Materials**

**Prognostic Significance of Combined PD-L1 Expression in Malignant and Infiltrating Cells in Hepatocellular Carcinoma Treated with Atezolizumab and Bevacizumab**

**Running title: PD-L1 and atezolizumab plus bevacizumab treated HCC**

Jaejun Lee^1,2#^, Jae-Sung Yoo^3#^, Ji Hoon Kim^1,4^, Dong Yeup Lee^2^, Keungmo Yang^1,2^, Bohyun Kim^5^, Joon-Il Choi^5^, Jeong Won Jang^1,2^, Jong Young Choi^1,2^, Seung Kew Yoon^1,2^, Ji Won Han^1,2^*, Pil Soo Sung^1,2^*

^1^The Catholic University Liver Research Center, Department of Biomedicine & Health Sciences, College of Medicine, The Catholic University of Korea, Seoul, Republic of Korea

^2^Division of Hepatology, Department of Internal Medicine, Seoul St. Mary’s Hospital, College of Medicine, The Catholic University of Korea, Seoul, Republic of Korea

^3^School of Medicine, Kyungpook National University, Daegu, Republic of Korea

^4^ Division of Hepatology, Department of Internal Medicine, Uijeongbu St. Mary’s Hospital, College of Medicine, The Catholic University of Korea, Seoul, Republic of Korea

^5^Departmend of Radiology, Seoul St. Mary’s Hospital, College of Medicine, The Catholic University of Korea, Seoul, Republic of Korea

#Equal contribution

**Corresponding author:**

**Pil Soo Sung. MD. PhD**

Division of Gastroenterology and Hepatology, Department of Internal Medicine, College of Medicine, Seoul St. Mary’s Hospital, The Catholic University of Korea, 222 Banpo-daero, Seocho-gu, 06591 Seoul, Korea

Tel: +82-2258-2073 Fax: +82-2-3481-4025, E-mail: pssung@catholic.ac.kr

ORCID 0000-0002-5780-9607

**Ji Won Han. MD. PhD**

Division of Gastroenterology and Hepatology, Department of Internal Medicine, College of Medicine, Seoul St. Mary’s Hospital, The Catholic University of Korea, 222 Banpo-daero, Seocho-gu, 06591 Seoul, Korea

Tel: +82-2258-2073 Fax: +82-2-3481-4025, E-mail: tmznjf@catholic.ac.kr

ORCID 0000-0003-1456-1450

**A Table of Contents**

**Supplementary Tables**

Table S1. Inclusion and exclusion criteria

**Supplementary Figures**

Fig. S1. Dose-response curve for hazard ratio of survival outcomes depending on the PD-L1 level using restricted cubic spline curve analysis.

Fig. S2. Comparison of survival outcomes using CPS 5 as a cutoff value.

Fig. S3. Survival analysis in the subgroup of patients with viral etiologies.

**Table S1. Inclusion and exclusion criteria**

| Inclusion | Exclusion |
| --- | --- |
| 1) Patients diagnosed with unresectable HCC  2) Availability of histological data with immunohistochemical staining for PD-L1 in cell  3) Age ≥ 18 years  4) ECOG performance status ≤ 1; and  5) Patients who had at least one follow-up visit at the clinic after receiving AB treatment | 1) Patients with concurrent extrahepatic malignancies  2) Patients with severe liver dysfunction classified as Child-Pugh class C |

Abbreviations: HCC, hepatocellular carcinoma; PD-L1, programmed death-ligand 1; ECOG, Eastern Cooperative Oncology Group; AB, atezolizumab plus bevacizumab.

**Fig. S1. Dose-response curve for hazard ratio of survival outcomes depending on the PD-L1 level using restricted cubic spline curve analysis.** **(A)** **Overall survival, (B) Progression-free survival.** PD-L1 level of CPS 5 was set as a reference. Abbreviations: CI, confidence interval; HR, hazard ratio.


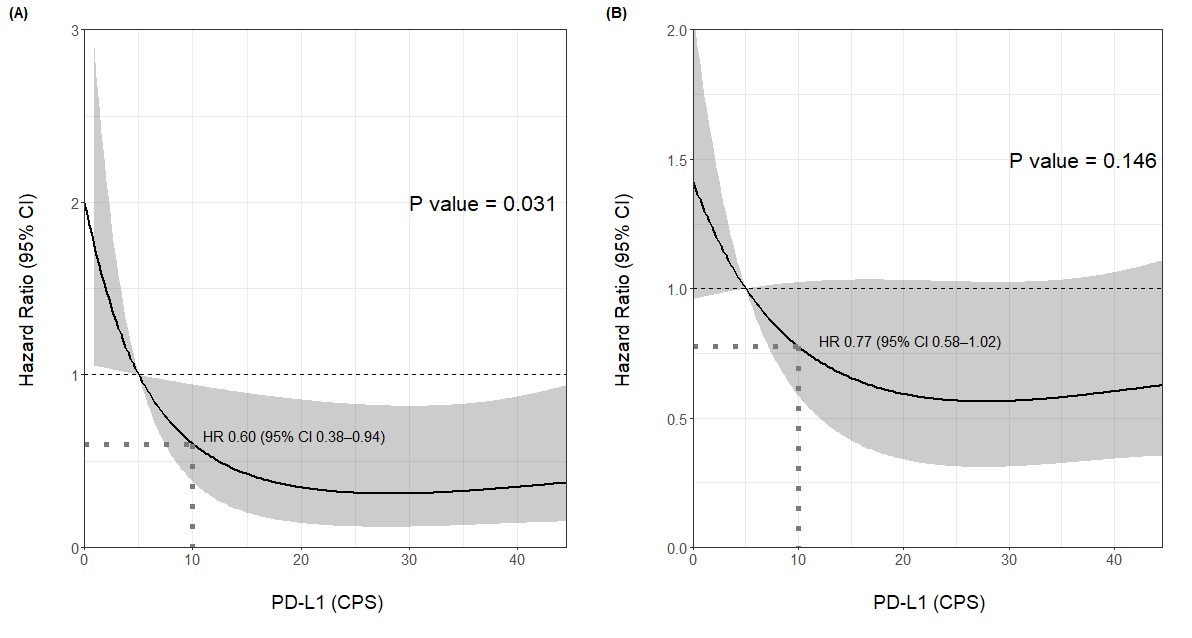


**Fig. S2. Comparison of survival outcomes using CPS 5 as a cutoff value. (A) Overall survival and (B) Progression-free survival.** CPS, combined positive score.


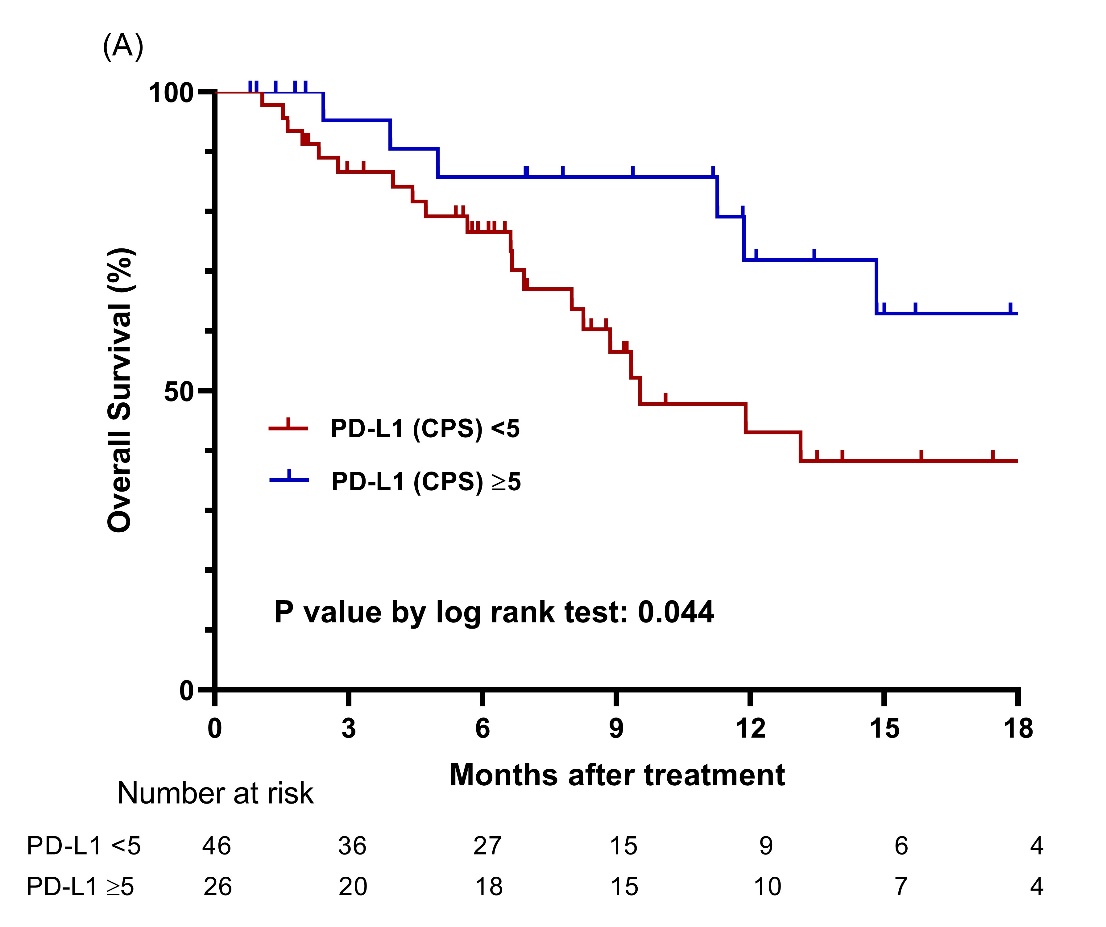


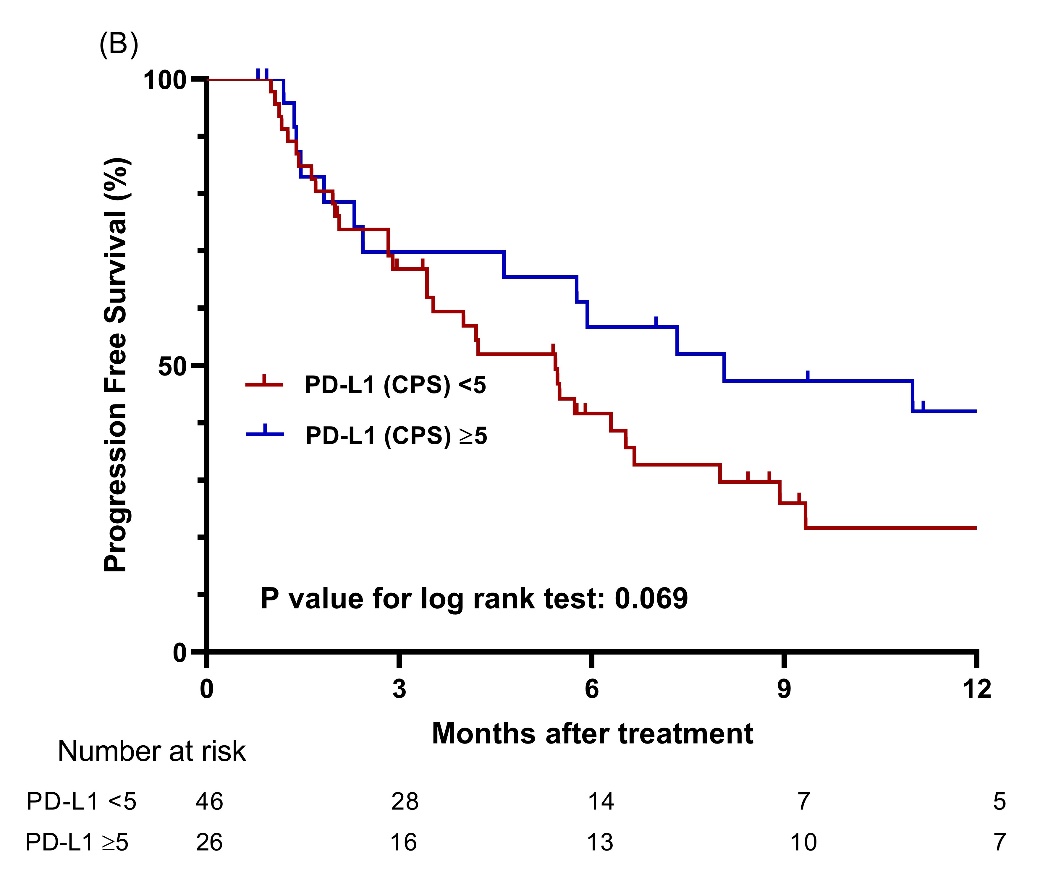


**Fig. S3. Survival analysis in the subgroup of patients with viral etiologies. (A) Overall survival and (B) Progression-free survival.** CPS, combined positive score


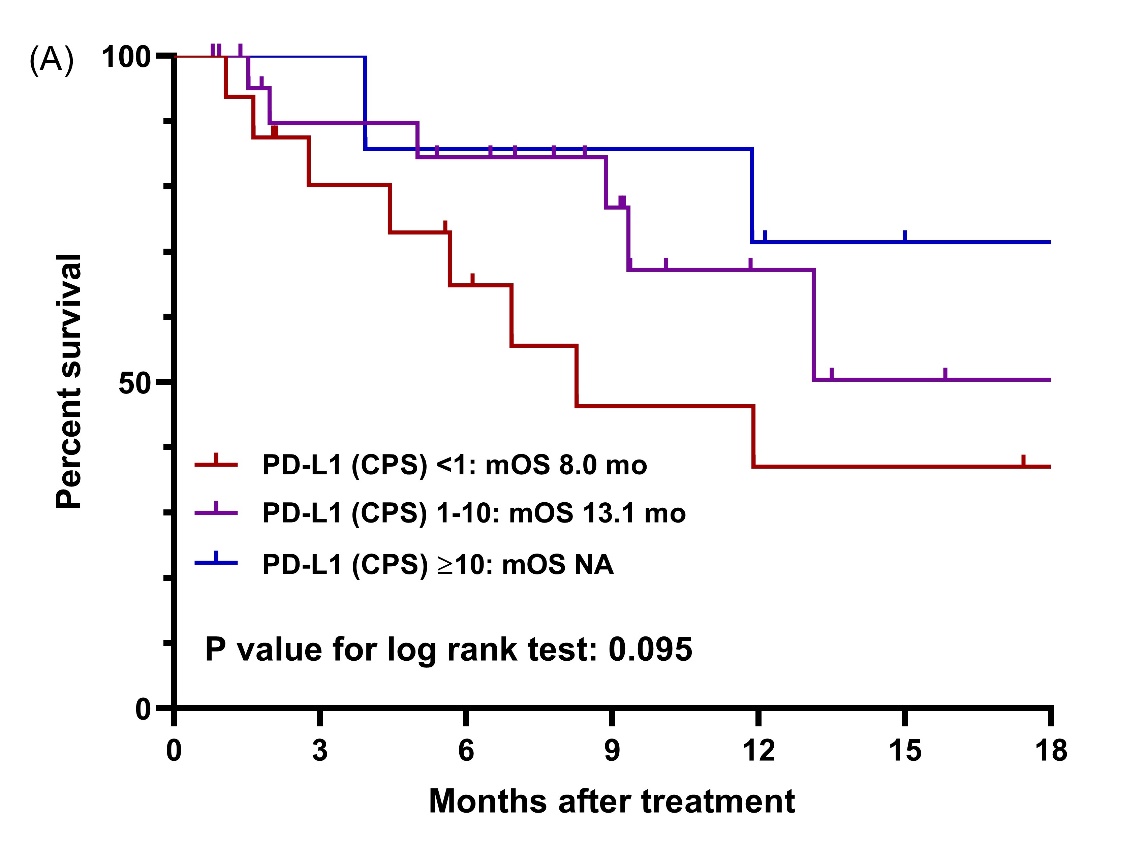


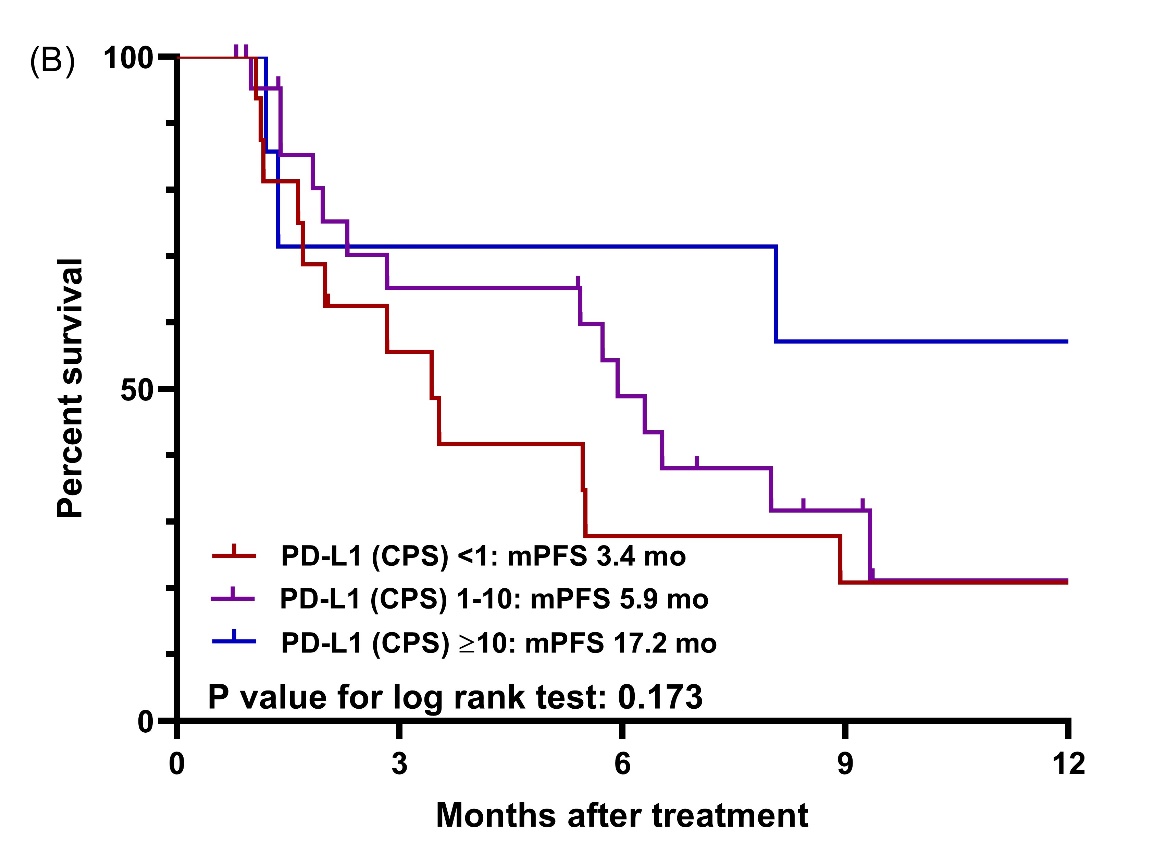

Supplement: Supplementary file 1 [file DataSheet1.docx]
